# Ambient Coarse Particulate Matter and Hospital Admissions in the Medicare Cohort Air Pollution Study, 1999–2010

**DOI:** 10.1289/ehp.1408720

**Published:** 2015-04-14

**Authors:** Helen Powell, Jenna R. Krall, Yun Wang, Michelle L. Bell, Roger D. Peng

**Affiliations:** 1 Department of Biostatistics, Johns Hopkins Bloomberg School of Public Health, Baltimore, Maryland, USA; 2 Department of Biostatistics, Harvard T.H. Chan School of Public Health, Boston, Massachusetts, USA; 3 School of Forestry and Environmental Studies, Yale University, New Haven, Connecticut, USA

## Abstract

**Background:**

In recent years a number of studies have examined the short-term association between coarse particulate matter (PM_10–2.5_) and mortality and morbidity outcomes. These studies, however, have produced inconsistent conclusions.

**Objectives:**

We estimated both the national- and regional-level associations between PM_10–2.5_ and emergency hospitalizations for both cardiovascular and respiratory disease among Medicare enrollees ≥ 65 years of age during the 12-year period 1999 through 2010.

**Methods:**

Using air pollution data obtained from the U.S. Environmental Protection Agency air quality monitoring network and daily emergency hospitalizations for 110 large urban U.S. counties assembled from the Medicare Cohort Air Pollution Study (MCAPS), we estimated the association between short-term exposure to PM_10–2.5_ and hospitalizations using a two-stage Bayesian hierarchical model and Poisson log-linear regression models.

**Results:**

A 10-μg/m^3^ increase in PM_10–2.5_ was associated with a significant increase in same-day cardiovascular hospitalizations [0.69%; 95% posterior interval (PI): 0.45, 0.92]. After adjusting for PM_2.5_, this association remained significant (0.63%; 95% PI: 0.38, 0.88). A 10-μg/m^3^ increase in PM_10–2.5_ was not associated with a significant increase in respiratory-related hospitalizations.

**Conclusions:**

We found statistically significant evidence that daily variation in PM_10–2.5_ is associated with emergency hospitalizations for cardiovascular diseases among Medicare enrollees ≥ 65 years of age. This association was robust to adjustment for concentrations of PM_2.5_.

**Citation:**

Powell H, Krall JR, Wang Y, Bell ML, Peng RD. 2015. Ambient coarse particulate matter and hospital admissions in the Medicare Cohort Air Pollution Study, 1999–2010. Environ Health Perspect 123:1152–1158; http://dx.doi.org/10.1289/ehp.1408720

## Introduction

Particle size is known to influence the deposition of airborne particulate matter (PM) within the respiratory tract. Currently, particles < 10 μm in aerodynamic diameter (PM_10_) and < 2.5 μm in aerodynamic diameter (fine, PM_2.5_) are both considered harmful to human health by the World Health Organization (WHO 2006). Governing bodies around the world, including the U.S. Environmental Protection Agency (EPA), currently monitor and regulate particulate matter at these metrics. However, it is difficult to disentangle the health effects associated with PM_10_ from those associated with PM_2.5_ because PM_10_ measurements consist largely of the finer PM_2.5_ particles (Brunekreef and Forsberg 2005).

A number of studies have estimated the health effects associated with coarse PM, which includes particles between 2.5 and 10 μm in aerodynamic diameter (PM_10–2.5_). Coarse PM is also referred to as “coarse thoracic PM” because the inhaled particles are deposited in the lower respiratory tract. PM_10–2.5_ particles are primarily crustal in nature (Chang et al. 2011), whereas PM_2.5_ particles are primarily generated by combustion processes (U.S. EPA 2009). However, the evidence evaluated from studies of PM_10–2.5_ has provided inconsistent conclusions and has thus led the 2009 Integrated Science Assessment from the U.S. EPA to determine the causal relationship between health outcomes and PM_10–2.5_ as “suggestive” (U.S. EPA 2009). As of January 2013, the National Ambient Air Quality Standards (NAAQS) did not include a standard for PM_10–2.5_ and instead retained the current standards for PM_10_ as a means of controlling for PM_10–2.5_ (U.S. EPA 2013).

A large national study of PM_10–2.5_ and hospitalizations for cardiovascular and respiratory diseases in the Medicare population was conducted in 2008 (Peng et al. 2008). That study found that daily changes in PM_10–2.5_ were positively correlated with daily cardiovascular hospitalizations but that this association was not statistically significant once it was adjusted for concurrent day PM_2.5_ concentrations. No statistically significant association was found between PM_10–2.5_ and respiratory hospitalizations. A key limitation of the study by Peng et al. (2008) was the limited sample size (and hence power), which included only data from 1999 to 2005. Because estimating PM_10–2.5_ concentrations requires measurements of PM_10_, which are typically measured on a less frequent 1-in-6 day schedule, data were not as abundant for that study as they were for previous national studies of PM_2.5_ alone (e.g., Bell et al. 2008a; Dominici et al. 2006).

The lack of clear national evidence on the health effects of PM_10–2.5_ and the continuing lack of a national ambient air quality standard specifically for this size fraction motivated the present study. From Medicare, a national social insurance program that guarantees access to health care for American citizens ≥ 65 years of age, we assembled a national database of cause-specific emergency hospitalizations among people in this age category. These data were linked with corresponding national databases of air pollution concentrations and weather information for 110 large urban communities within the United States spanning the 12-year period 1999–2010, with a sample size nearly double that in the Peng et al. (2008) study, which also examined this population. Using these national databases, we conducted a multisite time-series study to investigate the short-term association between PM_10–2.5_ and daily hospitalization for cardiovascular and respiratory diseases in an elderly population.

## Materials and Methods

**Figure 1:**
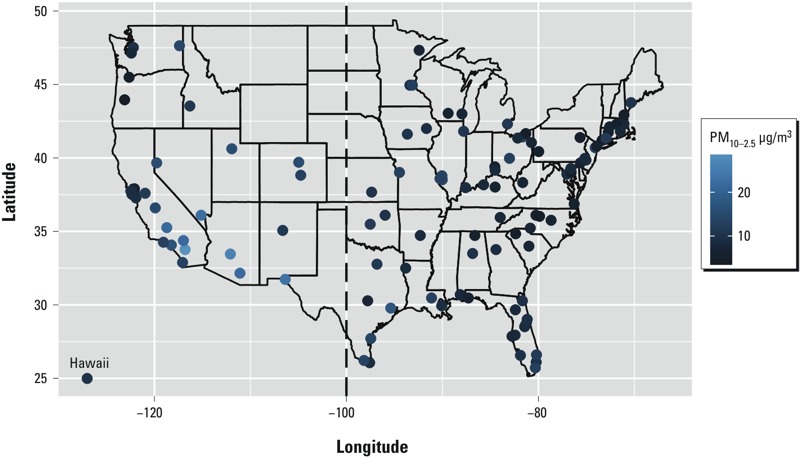
The location of each of the 110 U.S. counties and their median PM_10–2.5_ levels over all days for which data were available.

*Data*. The data presented here represent an extension of the Medicare Cohort Air Pollution Study (MCAPS) described previously (Dominici et al. 2006; Peng et al. 2008). For the study period 1 January 1999 through 31 December 2010, 110 U.S. counties were eligible for inclusion in this study. Counties were eligible for inclusion if they had > 20,000 Medicare enrollees ≥ 65 years of age in the year 2000 and had PM_10_ and PM_2.5_ recorded at collocated monitors for at least 200 days of the study period (1 January 1999 through 31 December 2010), with at least 90% of the measured PM_10_ concentrations greater in value than the collocated PM_2.5_ concentrations to ensure positive values of PM_10–2.5_, where PM_10–2.5_ is estimated by subtracting PM_2.5_ from PM_10_. In addition, the overdispersed Poisson log-linear model used to estimate county-level associations in the first stage of our analysis had to converge within a prespecified number of iterations when run using the data from a potentially eligible county. A total of 110 counties that met these criteria were included in our analysis (Figure 1). As described by Dominici et al. (2006), we classified counties into western and eastern regions according to their location relative to –100.00° longitude (*n* = 29 and 81 counties, respectively).

The health outcome of interest is the daily number of emergency hospital admissions for cardiovascular and respiratory diseases that can be derived from the billings claims of Medicare enrollees from the National Claims History Files. Medicare is a national social insurance program that guarantees access to health insurance for American citizens ≥ 65 years of age. As of 2012, 42.1 million people, approximately 94% of the American population in this age category, were enrolled in the Medicare program (Boards of Trustees, Federal Hospital Insurance and Federal Supplementary Medical Insurance Trust Funds 2013). The billings claim for each individual includes demographic and county-of-residence information in addition to the medical record. It is therefore possible to know the age of each claimant, which county they reside in, the date of their admission to the hospital, and their primary disease classification, which is given in accordance with the *International Classification of Diseases, Ninth Revision* (ICD-9).

We separately considered the broad outcomes of cardiovascular and respiratory disease, where the primary diagnosis at the time of admission to the hospital was used as the basis for inclusion into either category. We considered the same individual diagnoses as Peng et al. (2008), all of which have previously been associated with particulate matter (Bell et al. 2008a; Burnett et al. 1999; Lippmann et al. 2000). Specifically, we evaluated hospitalization for a primary diagnosis of cardiovascular outcomes (as a group and individually), including cerebrovascular events (ICD-9 codes 430–438), heart failure (HF; code 428), heart rhythm disturbances (HRD; codes 426–427), ischemic heart disease (IHD; codes 410–414 and 429), and peripheral vascular disease (PVD; codes 440–448). We also evaluated hospitalization for two respiratory outcomes (combined and individually): chronic obstructive pulmonary disease (COPD; codes 490–492) and respiratory tract infection (RTI, codes 464–466 and 480–487). For each county a daily time series of the hospitalization rates were constructed for both the individual diagnoses and the combined cardiovascular and respiratory outcomes. For the time period under consideration, there were a total of 6.37 million cardiovascular and 2.51 million respiratory emergency hospitalizations over the 110 counties.

The U.S. EPA employs a population-orientated air quality monitoring network that uses gravimetric methods to analyze atmospheric pollutants. Both PM_2.5_ and PM_10_ are currently monitored by this network (http://1.usa.gov/1oJDcyz). Therefore, we derived an indicator of PM_10–2.5_ by subtracting the daily measured PM_2.5_ concentrations from the same-day PM_10_ concentrations at locations with collocated monitors, as routinely performed by the U.S. EPA (Vanderpool et al. 2004) and commonly used in epidemiological studies. For counties with more than a single pair of collocated monitors, we applied a 10% trimmed mean (Samet et al. 2000) to obtain a single estimate of PM_10–2.5_ for each day of the study and protect against outliers. If there were fewer than 10 collocated monitors, we instead dropped the minimum and maximum values for that day; if only two estimates were available, the average was used. This method has been used in numerous previous air pollution studies (e.g., Dominici et al. 2006; Peng et al. 2008). In addition, we obtained daily temperature and dew-point-temperature data for each county from the National Climatic Data Center (EarthInfo, Inc. 2014) because both are potential confounders of the association between health outcomes and air pollution exposures.

*Analyses*. To estimate both regional (eastern vs. western United States) and national associations between PM_10–2.5_ and county-level hospitalization rates, we used a two-stage Bayesian hierarchical model. Adjustments were made for PM_2.5_, weather, seasonal, and long-term trends. In addition to the single-day exposure lags of 0, 1, and 2 days, which were originally considered by Peng et al. (2008), we also looked at an exposure lag of 3 days. We included this additional lag because a number of recent studies have found a significant association between PM_10–2.5_ and a health outcome at this longer 3-day lag period (Tecer et al. 2008) or at an average exposure level that includes days 0 to 3 (Bell et al. 2008a; Qiu et al. 2012, 2013). Due to the small subset of counties with daily data, we did not, however, include a 3-day moving average lag.

In the first stage, we stratified the time series by age, creating two categories: one for those Medicare enrollees between 65 and 74 years of age, and one for those ≥ 75 years. We used an overdispersed Poisson log-linear model to estimate county-level associations between PM_10–2.5_ and hospitalizations. This model included an offset of the natural logarithm of the number of people at risk on a given day in that county, taken to be the total number of Medicare enrollees for that county on that day; a separate intercept for each age category to account for differing baseline hospitalizations rates in each of the age categories (65–74 years and ≥ 75 years); an indicator for the day of the week; an indicator for those ≥ 75 years of age; smooth functions of the current day’s temperature and the mean of the previous 3 days’ temperatures, both of which used 6 degrees of freedom; smooth functions of the current day’s dew point temperature and the mean of the previous 3 days’ dew point temperatures, both of which used 3 degrees of freedom; a smooth function of calendar time, with 8 degrees of freedom per year for cardiovascular admissions and 12 per year for respiratory admissions (respiratory admissions are more strongly seasonal than cardiovascular admissions); a smooth function of time interacting with the indicator for age to capture the varying long-term time trends, with 1 degree of freedom per year; and the daily PM_10–2.5_ concentrations at the given lag. Each of the smooth functions was included as a natural cubic spline. The degrees of freedom per year for the smooth function of time were chosen so that longer-term and seasonal fluctuations in PM_10–2.5_ and hospitalizations were removed, leaving only shorter-term fluctuations for estimating health effects. Degrees of freedom for the temperature and dew-point-temperature smooth functions were chosen to accommodate nonlinear relationships between these factors and the health outcomes (Curriero et al. 2002). In addition, a second model was also fit that included PM_2.5_ concentrations in addition to the variables listed above. Simultaneously including both PM_10–2.5_ and PM_2.5_ at the same lag allows us to adjust for the potential effect of PM_2.5_. This model was implemented in R statistical software, version 3.2.1 (R Core Team 2015) using the glm function, which uses only complete cases in the analysis.

At the second stage, we estimated the national- and regional-level associations between PM_10–2.5_ and hospital admissions using Bayesian hierarchical models within the tlnise package (Everson and Morris 2000). This allows us to combine the relative risk estimates across counties while accounting for within-county statistical error and between-county variability of the true relative risks. The county-specific relative risks over all 110 counties were combined to produce a national-level estimate. Similarly, regional estimates for the western and eastern United States were produced by combining the county-specific relative risks for the 29 counties that lie to west and the 81 counties that lie to the east.

Statistical significance was assessed by the 95% posterior intervals (PIs), excluding the value of zero.

*Sensitivity analysis*. We assessed the sensitivity of the same-day national estimates for the cardiovascular and respiratory hospitalization rates with respect to the degrees of freedom that were used in the smooth functions of time, temperature, and dew point temperature. For both calendar time and temperature, we considered a range of degrees of freedom from 2 to 20; for dew point temperature, we considered a range from 1 to 10. The different choices for the degrees of freedom for each smooth function were considered independently of the other smooth functions.

## Results

Regionally, the daily admission rates were higher in the eastern United States compared with the western region for both cardiovascular and respiratory diseases (Table 1).

**Table 1:** Daily hospital admission rates per 100,000 [median (25th–75th percentiles)] for the period 1999–2010 for both the individual and the overall cardiovascular and respiratory health outcomes.

Outcome	All counties	Western	Eastern
Cardiovascular disease	1.85 (1.61–2.10)	1.43 (1.24–1.66)	1.99 (1.73–2.25)
Cerebrovascular disease	0.41 (0.37–0.45)	0.34 (0.29–0.39)	0.43 (0.39–0.48)
Heart failure	0.52 (0.45–0.59)	0.37 (0.31–0.43)	0.57 (0.50–0.65)
Heart rhythm disturbances	0.34 (0.27–0.36)	0.26 (0.22–0.29)	0.36 (0.29–0.39)
Ischemic heart disease	0.52 (0.40–0.63)	0.41 (0.32–0.52)	0.55 (0.43–0.67)
Peripheral vascular disease	0.08 (0.05–0.09)	0.05 (0.04–0.07)	0.08 (0.05–0.10)
Respiratory disease	0.67 (0.57–0.82)	0.57 (0.46–0.73)	0.71 (0.61–0.86)
COPD	0.23 (0.20–0.28)	0.17 (0.14–0.22)	0.25 (0.22–0.30)
Respiratory tract infections	0.44 (0.37–0.55)	0.40 (0.32–0.52)	0.46 (0.39–0.57)

During the study period, median PM_10–2.5_ concentrations were lower for counties in the east compared with counties in the west, whereas the opposite pattern was observed for PM_2.5_ (Figure 1, Table 2). The schedule for measuring PM_10_ and PM_2.5_ in each county is not always daily, and in some counties it may be as infrequent as once in every 6 days for PM_10_ and once in every 3 days for PM_2.5_. Therefore, there are a limited number of days for which PM_10–2.5_ can be estimated. Over all counties, the median number of days for which it was possible to estimate PM_10–2.5_ was 681, with 25th and 75th percentiles of 515 and 1,188, respectively. Both temperature and dew point temperature were higher in the eastern United States (Table 2).

**Table 2:** Levels of coarse particulate matter (PM_10–2.5)_, fine particulate matter (PM_2.5_), temperature and dew point temperature for the period 1999–2010.

Exposure	Median (25th–75th percentile)
PM_10–2.5_ (μg/m^3^)
All counties	12.78 (9.94–15.84)
Western counties	17.38 (13.07–21.99)
Eastern counties	9.77 (6.73–12.90)
PM_2.5_ (μg/m^3^)
All counties	12.06 (10.12–14.22)
Western counties	10.30 (8.24–13.49)
Eastern counties	12.17 (9.84–14.88)
Temperature (°F)
All counties	61.35 (48.56–73.76)
Western counties	59.21 (49.61–70.87)
Eastern counties	62.03 (48.45–74.09)
Dew point temperature (°F)
All counties	47.31 (36.48–59.15)
Western counties	40.54 (35.06–47.49)
Eastern counties	49.98 (37.25–63.11)

**Figure 2:**
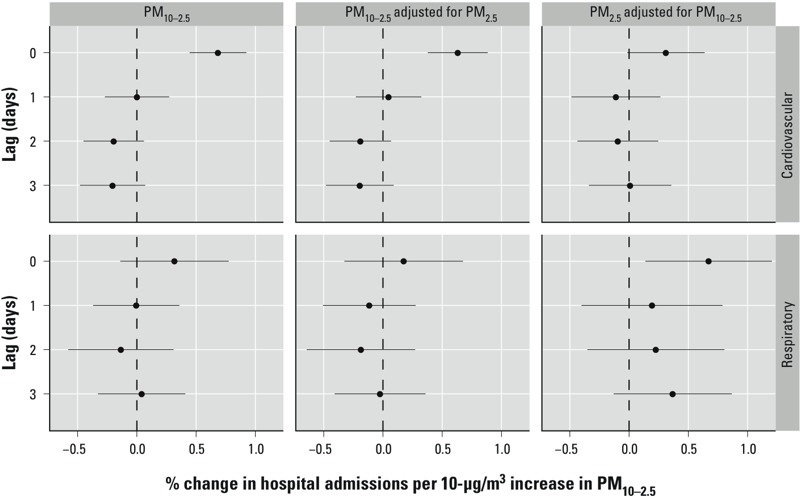
Estimated national-level associations and 95% PIs between cardiovascular and respiratory disease admissions and a 10-μg/m^3^ increase in PM_10–2.5_.

**Figure 3:**
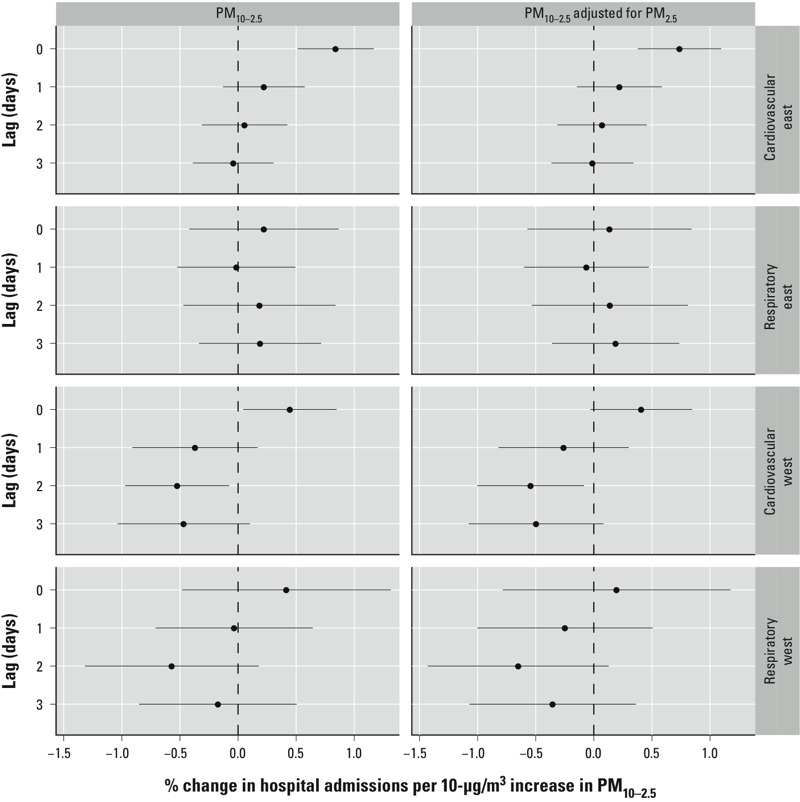
Estimated regional-level associations and 95% PIs between cardiovascular and respiratory disease admissions and a 10-μg/m^3^ increase in PM_10–2.5_.

**Figure 4:**
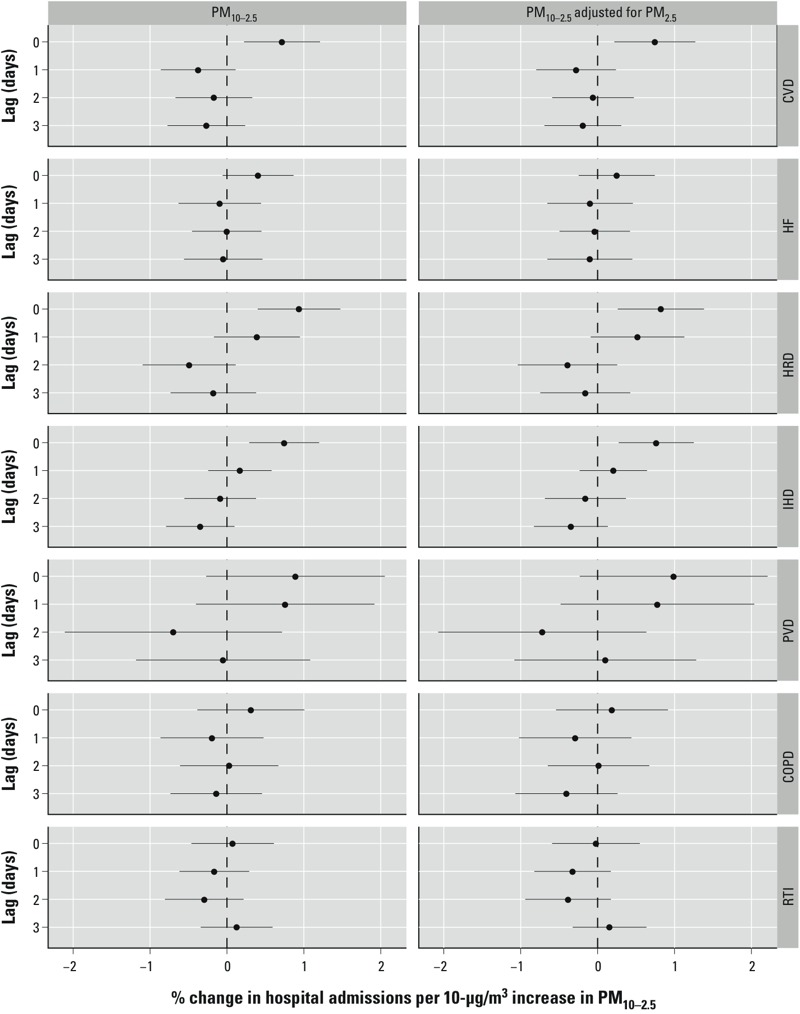
Estimated national-level associations and 95% PIs between cause-specific disease and a 10-μg/m^3^ increase in PM_10–2.5_. Abbreviations: COPD, chronic obstructive pulmonary disease; CVD, cerebrovascular disease; HF, heart failure; HRD, heart rhythm disturbances; IHD, ischemic heart disease; PVD, peripheral vascular disease; RTI, respiratory tract infection.

The results of both the national (Figures 2 and 4) and regional (Figure 3) level associations are presented as the percentage change in the number of emergency hospitalizations for a 10-μg/m^3^ increase in coarse particulate matter, with associated 95% PIs. Results are shown for lags 0, 1, 2, and 3 days for both the single-pollutant (only PM_10–2.5_ is included) and two-pollutant (PM_10–2.5_ and PM_2.5_ are jointly included) models.

Under both the single-pollutant and two-pollutant models, we found that a 10-μg/m^3^ increase in PM_10–2.5_ was associated with a statistically significant increase in cardiovascular hospitalizations on the same day as exposure, with increases of 0.69% (95% PI: 0.45, 0.92) and 0.63% (95% PI: 0.38, 0.88), respectively, under each model (Figure 2). Exposure at a lag of 1, 2, or 3 days was not significantly associated with the number of cardiovascular admissions under either model. Respiratory hospitalizations were not significantly associated with PM_10–2.5_ on the same day or any of the previous 3 days of exposure under either the single-pollutant or two-pollutant model (Figure 2). After adjusting for PM_10–2.5_, a 10-μg/m^3^ increase in PM_2.5_ was associated with a significant increase in respiratory hospitalizations on the same day (0.67%; 95% PI: 0.14, 1.21), whereas the association with cardiovascular admissions was not significant (0.31%; 95% PI: –0.02, 0.64).

A 10-μg/m^3^ increase in PM_10–2.5_ was positively associated with cardiovascular admissions on the same day in both eastern (0.84%; 95% PI: 0.51, 1.17) and western counties (0.44%; 95% PI: 0.04, 0.85) (Figure 3). After adjusting for PM_2.5_, the association remained statistically significant for the eastern (0.74%; 95% PI: 0.38, 1.10) but not the western region (0.41%; 95% PI: –0.03, 0.85). Under both the single- and two-pollutant models, exposure at a lag of 1, 2, or 3 days was not associated with a significant change in cardiovascular admissions in the eastern United States. In the west, PM_10–2.5_ was negatively associated with both cardiovascular and respiratory admissions at these lags under both models. At an exposure lag of 2 days, this negative association was significant for cardiovascular admissions (single-pollutant model, –0.52%; 95% PI: –0.97, –0.08 and two-pollutant model, –0.54%, 95% PI: –1.00, –0.08). A 10-μg/m^3^ increase in PM_10–2.5_ was not significantly associated with respiratory admissions in either the eastern or western United States at any exposure lag. These estimates and associated PIs are less precise, as demonstrated by the wider intervals, than those for the cardiovascular outcome because of the small number of respiratory admissions (Figure 3).

When examining the subcategories of cardiovascular admissions, a 10-μg/m^3^ increase in PM_10–2.5_ was associated with a significant increase in cerebrovascular disease [0.72% (95% PI: 0.22, 1.21) and 0.74% (95% PI: 0.22, 1.27)], heart rhythm disturbances [0.94% (95% PI: 0.40, 1.48) and 0.82% (95% PI: 0.26, 1.38)], and ischemic heart disease [0.74% (95% PI: 0.29, 1.20) and 0.76% (95% PI: 0.27, 1.25)] admissions on the same day (Figure 4) under both the single- and two-pollutant models, respectively. This increase in PM_10–2.5_ was also associated with a nonsignificant rise in same-day heart failure (0.40%; 95% PI: –0.06, 0.87) and peripheral vascular disease (0.89%; 95% PI: –0.27, 2.05) admissions under the single-pollutant model. Compared with heart failure, peripheral vascular disease was more strongly associated with same-day PM_10–2.5_; however, this estimated association was relatively imprecise because of the small number of admissions for this disease. A 10-μg/m^3^ increase in PM_10–2.5_ was not significantly associated with hospital admissions due to COPD [0.31% (95% PI: –0.39, 1.01) and 0.19% (95% PI: –0.54, 0.92)] or respiratory tract infections [0.07% (95% PI: –0.46, 0.61) and –0.02 (95% PI: –0.59, 0.55)] under either the single- or two-pollutant model, respectively. Exposure on previous days (lag 1, 2, or 3) was also not associated with significant change in hospital admissions for any of the individual subcategories under either model.

**Figure 5:**
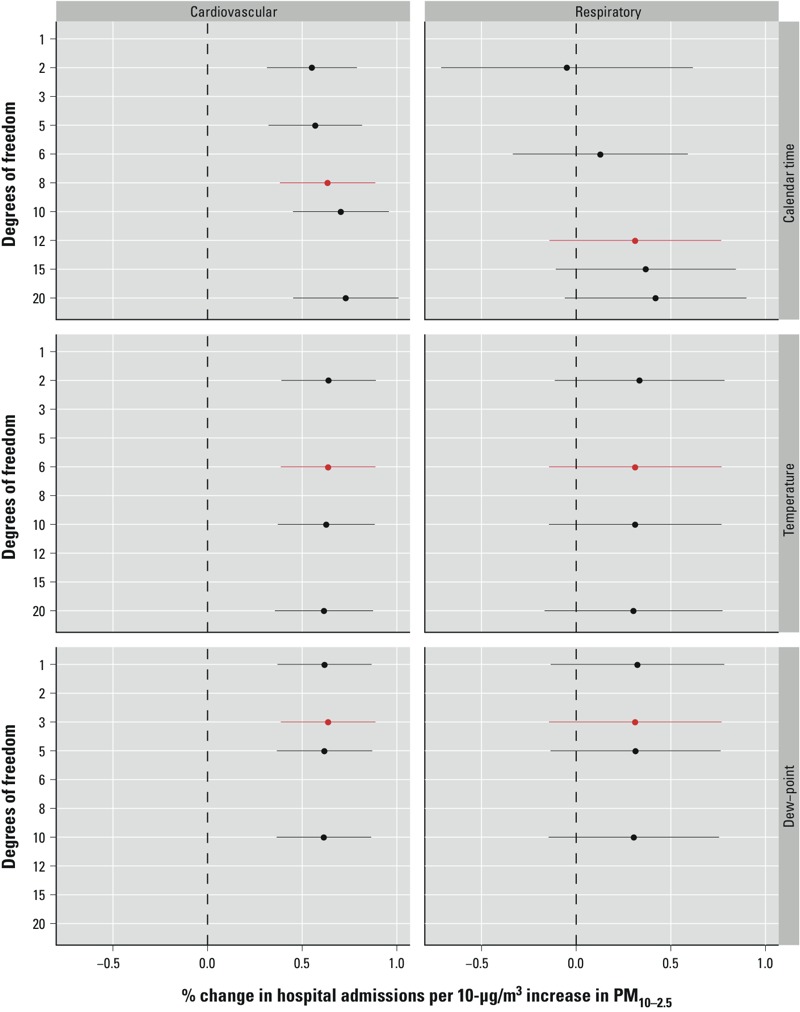
Estimated same-day national-level associations and 95% PIs between cardiovascular and respiratory disease admissions and a 10-μg/m^3^ increase in PM_10–2.5_ under varying degrees of freedom for the smooth functions of time, temperature, and dew point temperature. The estimates and PIs shown in red represent the degrees of freedom used in the final model.

We independently assessed the sensitivity of the same-day national average estimates with respect to the degrees of freedom used in the smooth functions of time, temperature, and dew point temperature under the single-pollutant model. From Figure 5 we can see that neither the cardiovascular nor respiratory hospitalization estimates showed substantial sensitivity to the choice of degrees of freedom for either the smooth functions of temperature or dew point temperature. The estimates are slightly more sensitive to the choice of degrees of freedom per year for the smooth function of time, particularly those relating to the respiratory admissions. However, given the very small number of degrees of freedom that we considered and the increasing width of the 95% PIs, this small amount of sensitivity is to be expected.

## Discussion

Using data on Medicare enrollees ≥ 65 years of age for the 12-year period, 1 January 1999 to 31 December 2010, we found that same-day exposure to PM_10–2.5_ was associated with increased cardiovascular hospitalizations, even after adjusting for PM_2.5_. We did not find evidence that PM_10–2.5_ was associated with respiratory hospitalizations, with or without adjustment for PM_2.5_.

Although previous studies have also found positive associations between cardiovascular hospitalizations and short-term exposure to PM_10–2.5_ (Brunekreef and Forsberg 2005; Peng et al. 2008; Qiu et al. 2013; Stafoggia et al. 2013), only one previous study examined these associations in a multicity U.S. study (Peng et al. 2008). Using a Medicare hospitalizations data set from 1999–2005, Peng et al. (2008) conducted a national study of 108 U.S. counties and found that a 10-μg/m^3^ increase in PM_10–2.5_ was associated with a 0.36% (95% PI: 0.05, 0.68) increase in cardiovascular admissions, but this association was not statistically significant after controlling for PM_2.5_ [0.25% (95% PI: –0.11, 0.60)]. We expanded the analysis of Peng et al. (2008) to include data for an additional 5 years (1999–2010) and found that a 10-μg/m^3^ increase in PM_10–2.5_ was associated with a statistically significant increase in cardiovascular hospitalizations, both without [0.69% (95% PI: 0.45, 0.92)] and with adjustment for PM_2.5_ [0.63% (95% PI: 0.38, 0.88)]. Despite using data and methods almost identical to those of Peng et al. (2008), our national estimates are greater in magnitude and, in the case of cardiovascular admissions, robust to adjustment for PM_2.5_. Possible explanations for this may include differences in the chemical composition of PM_10–2.5_ or differences in the overall health of the U.S. population from 1999 to 2010.

A 2005 literature review found evidence of associations between PM_10–2.5_ and increased respiratory morbidity (Brunekreef and Forsberg 2005), although the evidence from studies conducted since 2005 is more mixed (Host et al. 2008; Malig et al. 2013; Peng et al. 2008; Qiu et al. 2012; Stafoggia et al. 2013). Only one recent U.S. study found an association between PM_10–2.5_ and increased respiratory morbidity (Malig et al. 2013); however, 45% of the total cases included in that study were children under 18 years of age. In addition, that study focused on respiratory emergency department visits and might estimate a different underlying association between PM_10–2.5_ and respiratory morbidity than studies that focused on emergency respiratory hospitalizations. A study of six French cities found that PM_10–2.5_ was associated with increased respiratory hospitalizations in children < 14 years of age, but not in adults > 65 years (Host et al. 2008). As in another study of the U.S. Medicare population > 65 years of age (Peng et al. 2008), we did not find that short-term exposure to PM_10–2.5_ was significantly associated with respiratory hospitalizations.

Previous studies have also found short-term exposure to PM_10–2.5_ to be associated with increased mortality (Atkinson et al. 2010; Brunekreef and Forsberg 2005; López-Villarrubia et al. 2012; Malig and Ostro 2009; Zanobetti and Schwartz 2009). Although the magnitude of these mortality effect estimates were very varied, the largest U.S. study of PM_10–2.5_ and mortality (Zanobetti and Schwartz 2009) estimated effects similar in magnitude to those estimated in our study for cardiovascular hospitalizations.

In the present study, we found significant associations between PM_10–2.5_ and subcategories of cardiovascular disease hospitalizations, including cerebrovascular disease, heart rhythm disturbances, and ischemic heart disease. Heart failure and peripheral vascular disease, which are also subcategories of cardiovascular admissions, were not significantly associated with PM_10–2.5_. Other single-city studies have not found statistically significant associations between PM_10–2.5_ and hospitalizations due to cerebrovascular disease (Bell et al. 2008b; Halonen et al. 2009; Qiu et al. 2013), heart failure (Lippmann et al. 2000), or heart rhythm disturbances (Burnett et al. 1999; Halonen et al. 2009; Lippmann et al. 2000), although no national or regional studies have previously been conducted. Single-city studies have found associations with ischemic heart disease (Bell et al. 2008b; Burnett et al. 1999; Host et al. 2008; Lippmann et al. 2000; Qiu et al. 2013) and peripheral vascular disease (Burnett et al. 1999) hospitalizations. In our national-level study, we did not find evidence that PM_10–2.5_ was associated with hospitalizations due to COPD or respiratory tract infections, although some previous studies of single cities have found PM_10–2.5_ to be associated with hospitalizations due to COPD (Burnett et al. 1999; Chen et al. 2004; Qiu et al. 2012), combined asthma and COPD (Halonen et al. 2009), and respiratory infections (Burnett et al. 1999; Lippmann et al. 2000).

In our regional analysis, we found that PM_10–2.5_ was more strongly associated with cardiovascular hospitalizations in the eastern United States than in the western region. The regional differences in estimated health effects of PM_10–2.5_ may be explained by regional differences in the chemical composition of PM_10–2.5_, which varies with the sources of PM_10–2.5_, including sea spray, road dust, erosion, and bioaerosols (U.S. EPA 2009). Whereas the concentration of PM_10–2.5_ was higher in the west compared with the east, the chemical constituents present in PM_10–2.5_ may be more toxic in the east. Because the chemical composition of PM_10–2.5_ is not measured at the national scale in the United States, we cannot determine whether regional differences in estimated health effects of PM_10–2.5_ are attributable to chemical composition in this study. Regional differences in estimated health effects may also be driven by differences in personal exposure attributable to differences in air conditioning use or time spent outdoors. We estimated some negative associations between cardiovascular admissions and PM_10–2.5_ in the west at longer exposure lags. One possible explanation for such an observation is the presence of a short-term displacement of hospitalizations over the course of a few days, similar to what has occasionally been observed with mortality outcomes (Dominici et al. 2003).

Some previous studies outside the United States have found effect estimates for PM_10–2.5_ that were similar in magnitude to estimated effects for PM_2.5_ (Host et al. 2008; Bell et al. 2008b; Halonen et al. 2009; Stafoggia et al. 2013). Although we did not estimate associations for PM_2.5_ in this study, previous national-level studies of PM_2.5_ have estimated larger effect magnitudes for cardiovascular hospitalizations than we found in this study of PM_10–2.5_ (Dominici et al. 2006; Peng et al. 2008; Zanobetti et al. 2009). In contrast with previous studies of the health effects of PM_10–2.5_ (Peng et al. 2008; Qiu et al. 2012, 2013; Stafoggia et al. 2013), we found that the estimated associations between PM_10–2.5_ and hospitalizations were only slightly decreased after adjusting for PM_2.5_.

*Limitations*. In the United States, there is no national monitoring system for PM_10–2.5_, and most studies use the indirect method of taking the difference between PM_10_ and PM_2.5_ to estimate PM_10–2.5_ concentrations. This indirect approach leads to more measurement error than if we monitored PM_10–2.5_ directly because of the measurement error present in the observations of both PM_10_ and PM_2.5_. We did not have daily measurements of PM_10–2.5_ in every county because of the monitoring schedules for PM_2.5_ and PM_10_; therefore, we needed to use different subsets of the hospitalization data when considering different exposure lags for PM_10–2.5_. However, because our hospitalization data set was very large, it is unlikely that the differences observed between the exposure lags were driven by the lack of daily PM_10–2.5_ concentrations.

Because we were unable to obtain daily concentrations of PM_10–2.5_ for every county in our study, we were unable to examine multiday effects of PM_10–2.5_ using distributed lag models (Schwartz 2000; Welty and Zeger 2005) or an exposure averaged over multiple days. Previous studies have found larger associations between PM_10–2.5_ and hospitalizations using the average PM_10–2.5_ concentration over 0–2 or 0–3 days preceding hospitalization (Bell et al. 2008b; Chen et al. 2004; Qiu et al. 2013). If the effect of PM_10–2.5_ on hospitalizations extends for multiple days, our study may underestimate associations by using single-day exposure lags for PM_10–2.5_.

To decrease the impact of outlying monitor values in our analysis, we used a 10% trimmed mean to estimate the concentration of PM_10–2.5_ within a county for days when multiple measurements of PM_10–2.5_ could be estimated. However, PM_10–2.5_ is a spatially heterogeneous pollutant (U.S. EPA 2009), and this approach may not sufficiently adjust for spatial misalignment error introduced by observing PM_10–2.5_ at ambient monitors. A previous study of PM_10–2.5_ did not find that estimated health effects differed substantially between using a 10% trimmed mean and using a more complex measurement error model for PM_10–2.5_ (Chang et al. 2011).

Most studies of the associations between PM_10–2.5_ and hospitalizations have been conducted outside the United States (Brunekreef and Forsberg 2005). One source of PM_10–2.5_ outside the United States is dust storms (Bell et al. 2008b; de Longueville et al. 2013; Karanasiou et al. 2012), which are not as common in the eastern United States and may lead to differences in the chemical composition of PM_10–2.5_ between the United States and other countries. Our study results may not be comparable to studies outside the United States because of other characteristics, such as differences in personal exposure, differences in health-care systems, or differences in the health profile of the population under consideration. Our study was limited to U.S. adults ≥ 65 years of age enrolled in Medicare and may not be generalizable to other, younger populations or populations outside the United States.

## Conclusion

In a national-level study of the U.S. Medicare population ≥ 65 years of age, we found that short-term exposure to PM_10–2.5_ was associated with increased same-day cardiovascular hospitalizations. This association between PM_10–2.5_ and cardiovascular hospitalizations remained statistically significant after adjusting for same-day concentrations of PM_2.5_. Although previous studies have been inconsistent, in this study, using the longest time frame yet for examining the short-term health effects of PM_10–2.5_, we provided statistically significant evidence that short-term increases in the coarse fraction of PM are harmful to human health. Our results indicate that a national monitoring network for PM_10–2.5_ may be necessary to track associations between PM_10–2.5_ and adverse health outcomes.
